# Cost-Effectiveness of Molecularly Guided Treatment in Diffuse Large B-Cell Lymphoma (DLBCL) in Patients under 60

**DOI:** 10.3390/cancers14040908

**Published:** 2022-02-12

**Authors:** Dean A. Regier, Brandon Chan, Sarah Costa, David W. Scott, Christian Steidl, Joseph M. Connors, Aly Karsan, Marco A. Marra, Robert Kridel, Ian Cromwell, Samantha Pollard

**Affiliations:** 1 Cancer Control Research, BC Cancer, Vancouver, BC V5Z 4C2, Canada; dregier@bccrc.ca (D.A.R.); brchan@bccrc.ca (B.C.); sarahecosta@gmail.com (S.C.); icromwell@cadth.ca (I.C.); 2 School of Population and Public Health, Faculty of Medicine, University of British Columbia, Vancouver, BC V6T 1Z3, Canada; 3 Centre for Lymphoid Cancer, BC Cancer, Vancouver, BC V5Z 1L3, Canada; dscott8@bccancer.bc.ca (D.W.S.); csteidl@bccancer.bc.ca (C.S.); jconnors@bccancer.bc.ca (J.M.C.); 4 Department of Medicine, Faculty of Medicine, University of British Columbia, Vancouver, BC V6T 1Z3, Canada; 5 Canada’s Michael Smith Genome Sciences Centre, BC Cancer, Vancouver, BC V5Z 4S6, Canada; akarsan@bcgsc.ca (A.K.); mmarra@bcgsc.ca (M.A.M.); 6 Institute of Medical Science, University of Toronto, Toronto, ON M5S 1A8, Canada; robert.kridel@uhn.ca

**Keywords:** cost-effectiveness analysis, diffuse large b-cell lymphoma, markov model, gene expression profiling, molecular subtyping

## Abstract

**Simple Summary:**

Diffuse large B cell Lymphoma (DLBCL) can be categorized into cell of origin (COO) subtypes. Genetic tests are used to detect which subtype of DLBCL a patient has. One subtype of DLBCL, activated B-cell like (ABC), is associated with a comparatively poorer prognosis. New evidence suggests that patients under 60 with ABC-DLBCL may benefit from ibrutinib added to their standard care treatment regimen. Genetic testing and treatments like ibrutinib can be costly to publicly funded healthcare systems. Here, we report a cost-effectiveness analysis of the addition of Ibrutinib to treat patients under the age of 60 with ABC-DLBCL. Our analysis found that the use of genetic testing to diagnose ABC-DLBCL and providing ibrutinib along with standard care treatment has a low to moderate probability of being cost-effective, depending on decision maker willingness to pay and the cost of the genetic test.

**Abstract:**

Background: Classifying diffuse large B-cell lymphoma (DLBCL) into cell-of-origin (COO) subtypes could allow for personalized cancer control. Evidence suggests that subtype-guided treatment may be beneficial in the activated B-cell (ABC) subtype of DLBCL, among patients under the age of 60. Methods: We estimated the cost-effectiveness of age- and subtype-specific treatment guided by gene expression profiling (GEP). A probabilistic Markov model examined costs and quality-adjusted life-years gained (QALY) accrued to patients under GEP-classified COO treatment over a 10-year time horizon. The model was calibrated to evaluate the adoption of ibrutinib as a first line treatment among patients under 60 years with ABC subtype DLBCL. The primary data source for efficacy was derived from published estimates of the PHOENIX trial. These inputs were supplemented with patient-level, real-world data from BC Cancer, which provides comprehensive cancer services to the population of British Columbia. Results: We found the cost-effectiveness of GEP-guided treatment vs. standard care was $77,806 per QALY (24.3% probability of cost-effectiveness at a willingness-to-pay (WTP) of $50,000/QALY; 53.7% probability at a WTP of $100,000/QALY) for first-line treatment. Cost-effectiveness was dependent on assumptions around decision-makers’ WTP and the cost of the assay. Conclusions: We encourage further clinical trials to reduce uncertainty around the implementation of GEP-classified COO personalized treatment in this patient population.

## 1. Introduction

Diffuse large B-cell lymphoma (DLBCL) represents 30–40% of all non-Hodgkin lymphomas [1]. Treatment with rituximab (Rituxan^®^; Roche; Basel, Switzerland) and a regimen of cyclophosphamide, doxorubicin, vincristine, and prednisone chemotherapy (R-CHOP) produces an overall five-year survival rate of 60% [2,3,4]. R-CHOP is also a cost-effective option with studies reporting incremental cost-effectiveness ratios (ICER) between $20,000 and $90,000 per quality-adjusted life-year (QALY) gained compared to CHOP alone [5,6]. Options for patients who experience R-CHOP treatment failure are limited and the majority will die from their disease [4]. 

Molecular profiling studies have demonstrated biological and clinical heterogeneity within DLBCL. At least two distinct cell-of-origin (COO) subtypes have emerged: germinal center B-cell-like (GCB) and activated B-cell-like (ABC) [4,7]. Patients with GCB and ABC subtypes experience different health outcomes. The overall survival of GCB patients treated with R-CHOP is between 65% and 85% compared to 45–69% for ABC patients [8,9,10,11]. Progression-free five-year survival for GCB and ABC is approximately 75% and 45%, respectively [10]. COO can be determined using immunohistochemistry-based (IHC) algorithms or gene expression profiling (GEP). IHC-based algorithms are less costly but limited by their binary nature (GCB or non-GCB subtypes), inter-laboratory/inter-observer variability, and a COO misclassification rate of up to 15% [9,12]. Conversely, GEP has greater accuracy but is more costly and requires a fresh frozen biopsy.

Evidence suggests that the efficacy of novel therapeutic agents is affected by molecular subtype [13,14,15,16]. A Phase III randomized controlled trial of previously untreated, non-GCB DLBCL patients (the “PHOENIX” trial, ClinicalTrials.gov: NCT01855750) found that the addition of ibrutinib (Imbruvica^®^; Janssen; Titusville, NJ, USA) to current standard care (R-CHOP) did not produce significant survival benefit for ABC-subtype patients, overall. In planned, post-hoc analyses, authors report that enrolled patients under 60 years of age experienced improved event-free survival (hazard ratio (HR) = 0.579; 95% confidence interval (CI) = 0.380–0.881), progression-free survival (HR = 0.556; 95% CI: 0.359–0.860), and overall survival (HR = 0.330; 95% CI 0.162–0.673) [17]. Notably, patients over 60 experienced a higher incidence of ibrutinib-related toxicity, resulting in reduced treatment administration which may have negatively impacted survival. 

In a retrospective analysis of PHOENIX trial biopsies, Wilson et al. (2021) investigated the presence of DLBCL genetic subtypes to further explore this finding [18]. Investigators found that survival benefit among patients receiving ibrutinib + R-CHOP was more pronounced within specific genetic subtypes (MCD and N1) [18,19], common in younger patient participants with ABC-COO. These findings offer preliminary evidence of a biological mechanism for the increased benefit of ibrutinib for patients under 60 with specific genetic subtypes common among the ABC subtype of DLBCL [18]. As suggested by emerging evidence, age-stratified evaluation of ibrutinib + R-CHOP for patients diagnosed with ABC subtype is a promising area for future investigation [17,18].

Targeted therapies carry high costs, and their effectiveness is uncertain [17]. Given the uncertainty of effect and expense of conducting additional randomized controlled trials, it is critical to examine whether a GEP-informed approach could be cost-effective with the confirmation of evidence produced by a new trial. We estimate the cost-effectiveness of ibrutinib in ABC subtype DLBCL among patients under 60, in a first-line setting, as an example of what might be accomplished using a GEP-informed approach to the treatment of DLBCL. In scenario analysis, we examine its application in the second line setting, post disease relapse. Alongside clinical evidence development for new health technologies and novel therapeutics, early-stage economic evaluations are critical to generating evidence to inform future reimbursement and access decision making.

## 2. Materials and Methods

We used decision analytic Markov modeling to characterize the expected cost-effectiveness of COO subtyping using GEP to guide treatment for DLBCL, in a cohort of patients under 60 years of age. Standard care was assumed to be R-CHOP. In the GEP-informed strategy, patients classified with ABC subtype received ibrutinib and R-CHOP. All other patients received R-CHOP alone. In a secondary analysis, we explored outcomes if patients in the GEP-informed strategy were receiving R-CHOP as primary treatment and their subtype was classified by the assay only if they experienced relapse. Patients with ABC subtype received ibrutinib in addition to standard post-relapse care. All other patients received standard post-relapse care. The HR and costs of ibrutinib were assumed to be equal in the second line setting as a baseline assumption.

We calculated mean costs and quality-adjusted life years (QALYs) for each treatment strategy. The primary outcome was the incremental cost-effectiveness ratio (ICER), calculated as the difference in mean costs divided by the difference in mean QALYs. We discounted costs and outcomes at a rate of 1.5% per year, consistent with Canadian guidelines [20]. The time horizon of the model was 10 years, representing the length of time when all members of the cohort had completed treatment and were either cured or had died. Each cycle represented six months of time. The perspective of the study was from the British Columbia Ministry of Health. Conditional on our model assumptions, statements about expected cost-effectiveness were based on a notional willingness to pay (WTP) threshold of $50,000 per QALY gained.

In response to emerging evidence illustrating a mechanism by which ABC-DLBCL patients may derive benefit through ibrutinib, we conducted a sensitivity analysis to account for the presence of genetic subtypes, MCD and N1. Informed by estimates reported by Wilson et al. (2021), we estimate that 67.7% of non-GCB subtyped patients under the age of 60 would be classified with MCD or N1 genetic subtypes and subsequently transition into the ibrutinib treatment arm [18].

### 2.1. Model Structure

**Figure 1:**
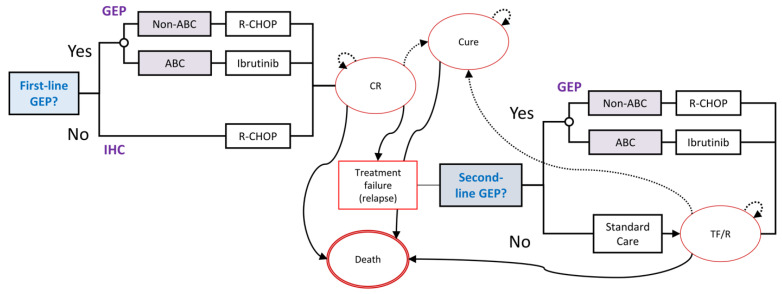
Decision model schematic. The Markov model simulates a hypothetical cohort of DLBCL patients over a ten-year time horizon. The Markov model health states are: complete response (CR), treatment failure or relapse (TF/R), cure or full remission, and death.

Our Markov model included time dependent transition probabilities and simulated a hypothetical cohort of newly diagnosed and previously untreated DLBCL patients, as outlined in Figure 1.

The model had four health states: complete response; treatment failure/relapse; full remission; and a terminal death state. The cohort begins in the complete response state after receiving first-line treatment. Conditional on experiencing disease relapse, patients move to the treatment failure/relapse state and subsequently receive second-line treatment. Patients may then respond to treatment and persist in the treatment/failure state or may experience treatment failure and die. After spending five years in the complete response or treatment failure/relapse states, patient cohort members transition to the cure state. For COO assignment, either at the time of first-line treatment (base case analysis) or treatment failure (in scenario analysis), we assumed the Lymph2Cx assay would be used [10]. This assay assigned patients into one of three groups: GCB, ABC, and unclassified DLBCL [10]. “Double-Hit” DLBCL (with *MYC* and *BCL2* and/or *BCL6* rearrangement) is identified using fluorescence *in situ* hybridization (FISH) testing. Those with GCB, Double-Hit, and unclassified DLBCL were characterized as non-ABC subtype.

### 2.2. Model Parameters

#### 2.2.1. Survival and Costs with Standard Care

For standard of care, we estimated the time to each event of relapse, death after relapse, death after complete response, or cure using secondary analysis of individual-level patient data for those under 60 years of age from the cohort reported by Scott et al. [9,10]. The cohort (Table 1) includes patients receiving care at BC Cancer who were treated with R-CHOP between 2000 and 2012 (*n* = 339; median follow-up of 6.5 years).

Transition probabilities were estimated for each COO subtype among patients under the age of 60 (*n* = 128). The proportion of the modeled cohort within each COO subtype was estimated from this same cohort, allowing for direct application of the subtype-specific treatment effect of ibrutinib to this subtype within this age cohort. Parametric coefficients were converted to time-dependent transition probabilities for each cycle [21]. 

Standard care treatment costs for DLBCL patients were derived from administrative healthcare records of a retrospective cohort of 751 patients previously treated at BC Cancer between 2004 and 2013 (median follow-up of 3.2 years), also described in Table 1. A full description of the costing methods is published elsewhere [22]. See Appendix B for a methodological and computational note on the generation of transition probabilities from stochastic survival data.

#### 2.2.2. Effectiveness and Cost of GEP-Informed Treatment

The incremental effectiveness of GEP-informed treatment was derived from the HRs published in the PHOENIX Trial [17]. The treatment HRs were sampled from a lognormal distribution of the mean published HR for event-free survival (EFS) and were subsequently applied to the coefficients for the hazard function derived in the retrospective survival analysis of the Scott et al. cohort [9]. The mean cost of the 20-gene GEP (Lymph2Cx) assay was taken from a previously conducted micro-costing study of high-throughput genomic assays at BC Cancer [23]. GEP-informed treatment cost was based on published estimates and recommended dosages for ibrutinib [24,25,26,27,28,29]. In estimating the cost effectiveness of GEP-informed second-line treatment, the cost of the assay was only applied to patients receiving second-line treatment.

#### 2.2.3. Health State Utilities

A systematic review of CHOP versus R-CHOP for DLBCL patients informed health state utility estimates for standard care [30]. We assumed the same utility values for GEP-informed R-CHOP treatment approaches. Utility estimates were sampled from a Beta distribution. All model parameters are summarized in Table 2.

#### 2.2.4. Cost-Effectiveness Analysis

The decision model was constructed in R (R Foundation for Statistical Computing, Vienna, Austria) and run using a Monte Carlo simulation process, drawing parameter estimates from distributions and simulating survival for 10,000 simulated cohorts of patients. The cycle-tree method was used for half-cycle correction [32]. All costs were converted to 2018 Canadian dollars ($CDN) using the BC-specific consumer price index for health and personal care [33]. Confidence intervals (95% CI) were estimated for costs and QALYs by bootstrapping10,000 Monte Carlo values 1000 times. This study was approved by the University of British Columbia-BC Cancer Research Ethics Board. All model input parameter estimates derived from the published literature can be found in Table 2.

## 3. Results

**Figure 2:**
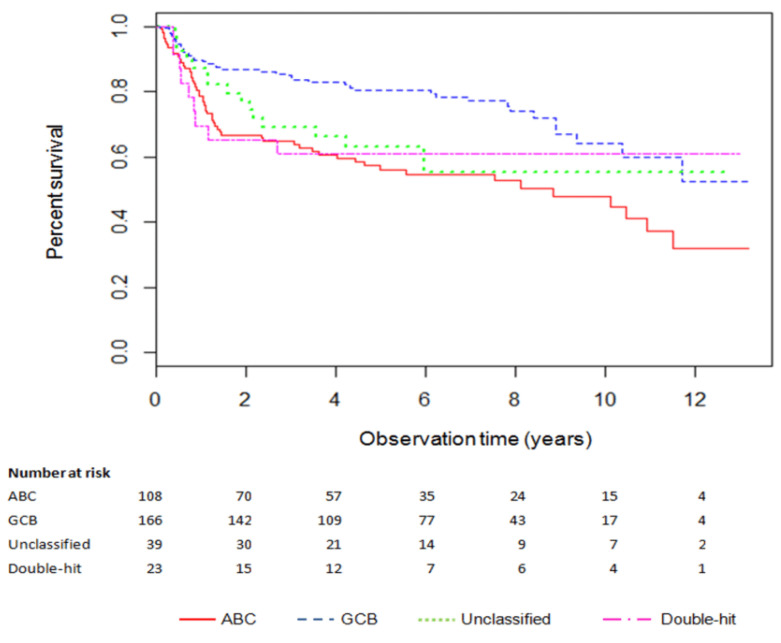
Kaplan-Meier plot of overall DLBCL survival by cell-of-origin subtype. Figure 2 legend: Figure 2 depicts the probability of survival for activated B-cell-like (ABC), germinal center B-cell-like (GCB), unclassified, and double-hit subtypes for patients who underwent cell of origin (COO) assignment but did not receive gene expression profiling (GEP)-informed treatment. The *y*-axis presents the proportion of patients alive at each time interval, and the *x*-axis is measured in years.

Kaplan–Meier survival curves for patients who underwent COO assignment but did not receive targeted therapy are shown in Figure 2, and model input parameters are shown in Table 2. 

The estimated mean cost for six cycles of standard care treatment (R-CHOP) was $29,120 (95% CI: 28,986–29,170). The baseline model assumed that the additional cost of ibrutinib in the GEP-informed strategy was $48,115 (95% CI: 27,368–68,861) over six cycles [24]. The mean per-case cost of a 20-gene GEP-based Lymph2Cx assay was estimated to be $438 (SE: 68) [23]. Total treatment-related costs were highest for the first six months following diagnosis, after which patients who achieve complete response or cure no longer require active treatment and accumulate costs for regular appointments and supportive medications (Appendix A, Table A1 and Table A2).

### Cost-Effectiveness of Treatment in Patients under 60 Years of Age

Costs and QALYs for the GEP-informed and standard care strategies are summarized for first- and second-line implementation of GEP-informed treatment in Table 3.

**Figure 3:**
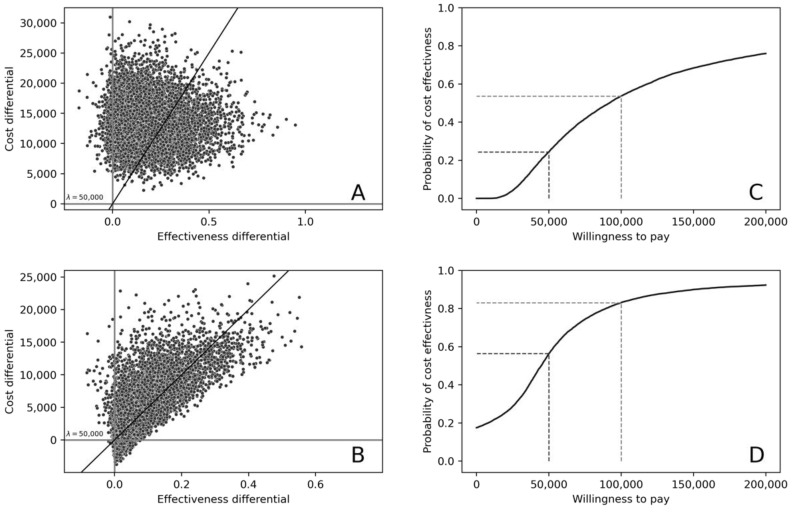
Cost-Effectiveness Acceptability Planes for (**A**) first- and (**B**) second line GEP-informed treatment of ABC subtype DLBCL, and Cost-effectiveness Acceptability Curves for (**C**) first- and (**D**) second line GEP-informed treatment of ABC subtype DLBCL. Figure 3 legend: (**A**,**B**) depict scatter plots of incremental cost-effectiveness ratios (ICERs) generated from the probabilistic analysis (*n* =  10,000 iterations). The upper right (North-West) quadrant of the cost effectiveness planes are shown, with ICERs demonstrating higher incremental costs alongside higher incremental effectiveness. In the first line treatment setting, 96% of simulated ICERs indicate higher incremental cost and higher incremental effectiveness (**A**), with 24% below a $50,000/QALY threshold (λ) which is the willingness-to-pay for an effectiveness gain. In the second line setting, respective proportions are 81% and 39%. Cost-effectiveness acceptability curves (**C**,**D**) illustrate the probability that GEP guided treatment is cost-effective at different levels of willingness-to-pay per additional QALY. The probability that first-line GEP-informed treatment was cost-effective at $50,000/QALY and $100,000/QALY were 24.3% and 53.7% respectively, and 56.3% and 83.1%, respectively, in second line treatment.

For first-line GEP-informed treatment, the mean incremental cost was $13,226 (95% CI: 5873–20,578), and mean incremental QALYs was 0.17 (95% CI: −0.09–0.43). The resulting ICER was $77,806. For second-line GEP-informed treatment, the incremental cost was $4270 (95% CI: −4621–13,161), and QALYs gained was 0.079 (95% CI: −0.07–0.23), resulting in an ICER of $53,909. The probability that first-line GEP-informed treatment was cost-effective at a WTP of $50,000/QALY and $100,000/QALY was 24.3% and 53.7% respectively. In second-line GEP-informed treatment, corresponding probabilities were 56.3% and 83.1%. Results are presented on the cost-effectiveness planes and as cost-effectiveness acceptability curves in Figure 3.

A threshold analysis identified the estimated hazard ratio at which GEP-informed therapy is no longer cost-effective. Threshold values of 0.43 and 0.67 were identified for thresholds of $50,000/QALY and $100,000/QALY respectively in first-line treatment. A univariate scenario analysis was conducted with an assay cost of $4500, representing the estimated cost of a commercial GEP test. The resulting ICERs were $98,222 (14.0% cost-effective at $50,000/QALY) and $57,786 (50.6% cost-effective at $50,000/QALY) for first- and second-line treatment respectively.

Additional scenario analysis estimated the probability of cost effectiveness under an assumption that 67.7% of ABC-subtyped patients would be further identified to have MCD or N1 genetic subtypes, subsequently initiating ibrutinib treatment (Table 4). The remaining 32.2% of patients were assumed to move into the standard care R-CHOP treatment arm.

**Figure 4:**
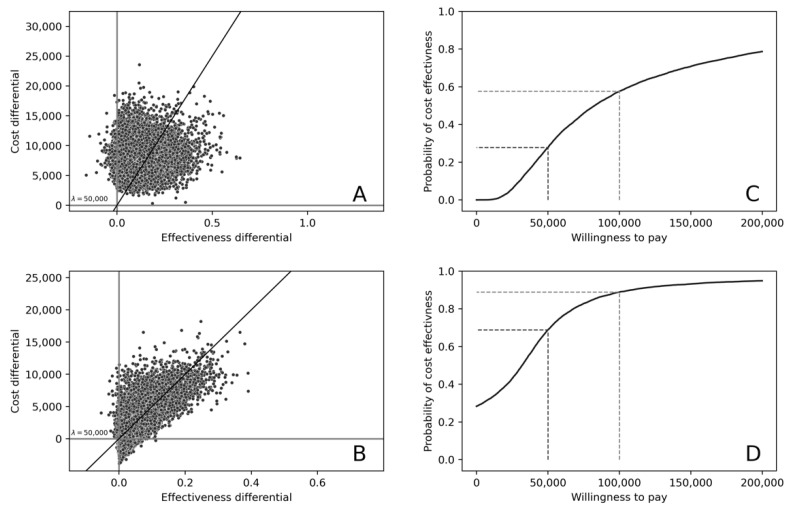
Cost-Effectiveness Acceptability Planes for (**A**) first- and (**B**) second line GEP-informed treatment of ABC subtype DLBCL, and Cost-effectiveness Acceptability Curves for (**C**) first- and (**D**) second line GEP-informed treatment of ABC subtype DLBCL. Figure 4 legend: (**A**,**B**) depict scatter plots of incremental cost-effectiveness ratios (ICERs) generated from the probabilistic analysis (*n* = 10,000 iterations). The upper right (North-West) quadrant of the cost effectiveness planes are shown, with ICERs demonstrating higher incremental costs alongside higher incremental effectiveness. In the first line treatment setting, 96% of simulated ICERs indicate higher incremental cost and higher incremental effectiveness (**A**), with 28% below a $50,000/QALY threshold (λ). In the second line setting, respective proportions are 70% and 41%. Cost-effectiveness acceptability curves (**C**,**D**) illustrate the probability that GEP guided treatment is cost-effective at different levels of willingness-to-pay per additional QALY. The probability that first-line GEP-informed treatment was cost-effective at $50,000/QALY and $100,000/QALY were 27.8% and 57.6% respectively, and 68.9% and 88.9%, respectively, in second line.

For first-line GEP-informed treatment, the mean incremental cost was $8386.55 (95% CI: 3150.32, 13,622.78), and mean incremental QALYs was 0.117 (95% CI: −0.065, 0.298). The resulting ICER was $71,706,37, as described in Table 4. For second-line GEP-informed treatment, the incremental cost was $2253.57 (95% CI: −4058.83, 8565.96), and QALYs gained was 0.0556 (95% CI: −0.05, 0.161), resulting in an ICER of $40,531,74. The probability that first-line GEP-informed treatment was cost-effective at $50,000/QALY and $100,000/QALY was 27.8% and 57.6% respectively, and 68.9% and 88.9% in second line treatment. Results are presented on the cost-effectiveness planes and as cost-effectiveness acceptability curves in Figure 4.

## 4. Discussion

Our analysis found that GEP-informed COO assignment in combination with ibrutinib + R-CHOP has low to moderate probability of being cost-effective among patients under the age of 60, depending on assumptions around decisions-makers’ willingness to pay and the cost of the assay. Our analysis estimated values of treatment effectiveness, health utility, and costs (including assay costs), regarding which the policy recommendation is likely to change (i.e., becomes no longer cost-effective on average). The model’s baseline findings were largely robust to decreases in univariate parameter uncertainty but were sensitive to assumption surrounding assay price.

Efforts to examine the cost effectiveness of subtype-guided treatment using GEP have been published elsewhere with lower ICERs found than are reported here [34,35]. The Chen and Staton et al. models [34,35] evaluated the potential use of first-line lenalidomide for non-GCB subtype lymphoma over a lifetime horizon, and derived survival for both standard care and GEP-informed patients from a phase II clinical trial. By contrast, our model, focused on patients under 60 years of age, was based on survival evidence from patients undergoing standard care and was adjusted to reflect treatment efficacy by using the published HR from a phase III trial.

Our work presents an early-stage cost effectiveness analysis of GEP-guided treatment with ibrutinib, among younger patients diagnosed with DLBCL. As evidence continues to emerge regarding clinical outcomes attributable to GEP-informed DLBCL management, our analysis provides information that decision-makers may use when considering the approval of such technologies.

### Limitations

**Figure A1:**
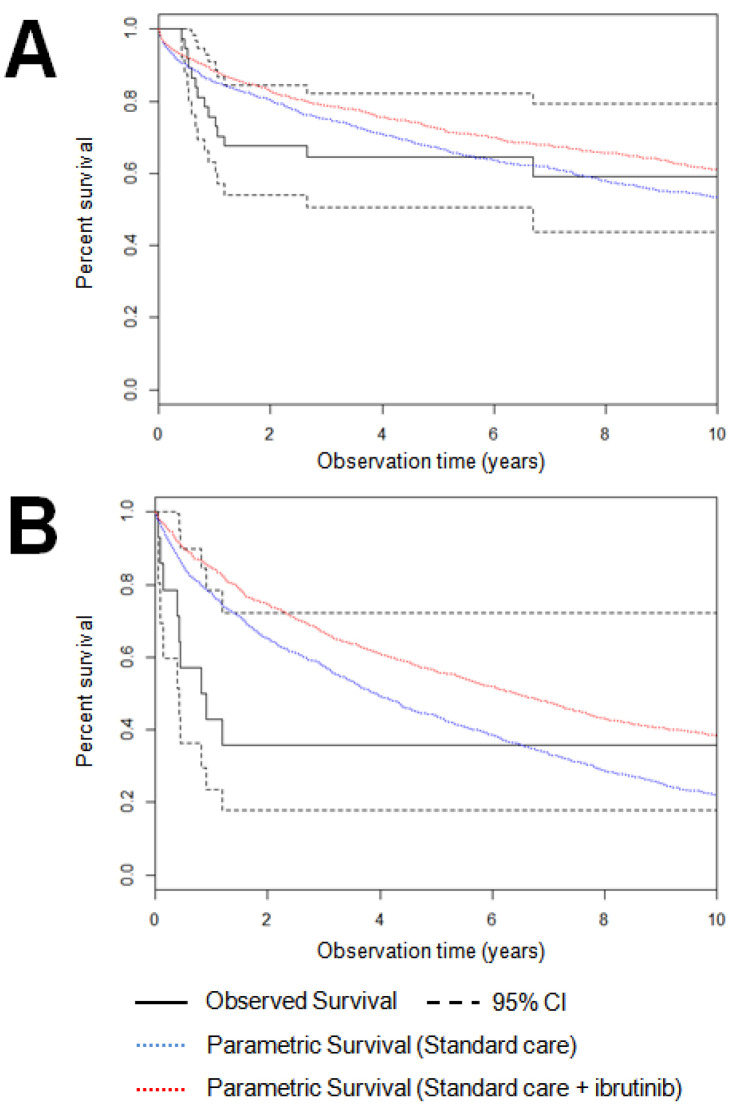
Observed and Parametric Survival for ABC subgroup patients under 60 for (**A**) Complete Response to Relapse, and (**B**) Relapse to Death.

There are limitations to our analysis that must be considered alongside the results. We assumed that the Lymph2Cx assay has perfect accuracy in determining ABC subtype, when test accuracy is an important source of uncertainty in economic evaluations of precision medicine [36,37]. It is worth noting that our survival analysis is based on observed data from Lymph2Cx subtypes, which mitigates the impact of this assumption. Second, our assumption of equivalent treatment efficacy of ibrutinib in both first- and second-line settings is not based on available evidence. Our parametric approach to calculating transition probabilities was also affected by a small sample size for the ABC subgroup (see Figure A1, Appendix A), meaning that these findings should be interpreted carefully. Thirdly, our baseline analysis presumes that quality of life estimates remain unchanged between comparator groups. This is primarily due to a lack of reliable estimates of utility for this disease group. Fourthly, our HRs for the efficacy ibrutinib for patients under 60 are not based on RCT evidence and as such should be interpreted with caution. We emphasize that confirmatory trials are needed to ensure efficacy. Fifthly, emerging evidence suggests that MCD and N1 genetic subtypes common among younger individuals with ABC-DLBCL may be driving the attributable benefit of ibrutinib. Our scenario analysis demonstrates the moderate probability of cost effectiveness of GEP-guided care where ibrutinib is reserved for patients carrying MCD and N1 subtypes of ABC-DLBCL, under the age of 60. As new evidence continues to emerge, there exists an ongoing need to generate early-stage cost effectiveness analyses to guide decision making. Finally, primary data used to inform model input parameters were generated using BC Cancer patient cohorts. BC Cancer is the provincial service provider for cancer prevention and treatment. A limitation to this approach is the use of British Columbia-specific cost and outcomes data to inform cost effectiveness estimates. This is because variation in costs and outcomes may vary between jurisdictions. To help account for uncertainty around parameter inputs, we conducted deterministic sensitivity analyses to account for the anticipated difference in relevant model parameters. To ensure jurisdiction specific relevance, we will make our decision model available for future evaluations to integrate updated and province-specific cost inputs.

## 5. Conclusions

Our analysis can be examined in light of ongoing discussions about optimal methods for COO assignment. The World Health Organization’s (WHO) classification of lymphoid neoplasms declared that COO characterization must be performed when diagnosing new DLBCL cancers, with at minimum two subtypes identified to allow for the potential use of targeted therapies in clinical practice [16]. There is also acknowledgement among researchers that COO classification should be used within prospective clinical trials [38]. Currently, in British Columbia, COO is performed on all newly diagnosed DLBCL cases using IHC-based methods. However, current evidence suggests these types of assays cannot be relied on to accurately inform clinical decision-making in terms of GEP-informed treatments. If GEP-based methods are to be more widely adopted in routine clinical practice, they must not only demonstrate that it is clinically beneficial to do so, but also economically feasible and practical as well. This paper generates evidence that will help to identify targets for clinical benefit and economic feasibility.

## Figures and Tables

**Table 1:** Descriptive characteristics for the BC Cancer retrospective cohort and the GEP-classified cohort.

	Study Cohorts		
Characteristics	Retrospective Cohort *n* = 751	GEP-Classified Cohort *n* = 339	GEP-Classified Cohort < 60 *n* = 128
Age at diagnosis			
Mean (range)	65 (18–93)	62 (16–92)	48 (16–59)
Age group			
0–19	<1%	<1%	2%
0–59	32%	37%	98%
60–69	28%	29%	NA
70–79	27%	26%	NA
80+	13%	7%	NA
Sex			
Male	58%	63%	68%
ECOG performance status at diagnosis			
0–1	62%	68%	69%
2–4	38%	31%	31%
missing	<1%	1%	0%
Stage of disease at diagnosis ^‡^			
Limited	29%	30%	30%
Advanced	71%	69%	70%
Other	-	1%	0%

GEP—gene expression profiling; ECOG PS, Eastern Cooperative Oncology Group Scale of Performance Status; NA = not applicable. ^‡^ Stage of disease defined according to Ann Arbor staging system.

**Table 2:** Model parameter inputs and data sources for costs.

Molecular Profiling Costs								
	Source	Mean			Standard Error			
GEP (Lymph2Cx) assay	Micro-costing study [23]	$438 *			$68			Gamma
Hans IHC-based algorithm	BC Cancer Estimates	$366 *			$54			
**Treatment costs**								
		**Mean**			**Standard error**			
GEP-informed treatment, first 6 cycles ^§^	Published estimates [24]	$48,115 *			$10,585			Gamma
**Treatment efficacy—Hazard ratio**								
R-CHOP		**Mean**			**Standard error**			
GEP-informed treatment (ABC subtype, <60 y)	Younes et al., 2019 [17]	−0.5465 *			0.01159			Normal
**Proportion of cell-of-origin subtype**								
		**Mean**		**Standard error**		**Alpha**		
ABC	Published estimates [9]	0.32 *		0.00255		108		Dirichlet
GCB		0.5 *		0.00205		166		
Unclassified		0.11 *		0.00281		38		
Double-hit		0.07 *		0.00294		23		
**Utilities**								
		**Mean**			**Standard error**			
Complete response, cure states	Published estimates (Knight et al., 2004) [30]	0.81 *			0.04			Beta
Treatment failure/relapse state		0.49 *			0.013			
**Transition probabilities—Weibull GLM regression coefficients**								
**From BC Cancer retrospective IHC/GEP-assigned cohort**								
		**Log (Scale)** **Log (SE)**			**Shape (ρ)** **SE**			
Complete Response to Treatment Failure/Relapse	ABC	4.888 *			−0.62 *			Normal
		1.048			0.251			
	GCB	5.334 *			−0.703			
		0.992			0.252			
	Unclassified	−4.87 *			−0.651			
		1.523			0.37			
	Double-hit	3.722 *			0.963 *			
		0.983			0.324			
Complete Response to Death	ABC	−5.354 *			−1.062			Normal
		1.811			0.664			
	GCB	−12.206			−0.039			
		7.21			0.875			
	Unclassified	−9.629 *			−0.045			
		3.624			0.483			
	Double-hit	−10.722			−0.017			
		6.843			0.846			
Treatment Failure/Relapse to Death	ABC	−3.339 *			−0.802 *			Normal
		0.99			0.3			
	GCB	−3.183 *			−0.835 *			
		0.924			0.29			
	Unclassified	−3.635			−0.519			
		1.385			0.344			
	Double-hit	−9.487			0.7			
		2.784			0.846			
Cure to Death	ABC	−11.801 *			0.309			Normal
		3.833			0.372			
	GCB	−16.508			0.675 *			
		4.066			0.274			
	Unclassified	−7.699 *			0.309			
		4.817			0.89			
	Double-hit	−16.508 *			0.675 *			
		4.066			0.274			

GEP—Gene expression profiling; IHC—Immunohistochemistry; R-CHOP—rituximab, cyclophosphamide, doxorubicin, vincristine, prednisone; ABC—Activated B Cell. * Denotes statistical significance at *p* < 0.05. ^§^ R-CHOP dosage recommendations as per BC Cancer chemotherapy protocols [31]. GEP-informed treatment dosage recommendations for base case as per NCT018557508 [28].

**Table 3:** Cost effectiveness results at varying assumptions of reductions in disease recurrence, baseline analysis.

Strategy	Cost	SD	ΔC	QALY	SD	ΔE	ICER
**First Line**							
Standard Care	$48,768	5781		5.443	1.603		
GEP-informed	$61,993	6488	$13,226	5.613	1.598	0.17	$77,806
**Second Line**							
Standard Care	$48,768	5782		5.443	1.603		
GEP-informed	$53,038	7813	$4270	5.522	1.593	0.079	$53,909

SD—standard deviation; QALY—quality-adjusted life year; ICER—incremental cost effectiveness ratio; ΔC = incremental cost (GEP-informed cost—standard care cost); ΔE = incremental effectiveness (GEP-informed mean QALY—standard care QALY).

**Table 4:** Cost effectiveness results at varying assumptions of reductions in disease recurrence, baseline analysis.

Strategy	Cost	SD	ΔC	QALY	SD	ΔE	ICER
**First Line**							
Standard Care	48,817.5	5797.82	8386.55	5.438	1.601	0.117	71,706.37
GEP-informed	57,204.05	5948.03		5.555	1.597		
**Second Line**							
Standard Care	48,817.5	5797.82	2253.57	5.438	1.601	0.056	40,531.74
GEP-informed	51,071.07	6945.73		5.493	1.594		

SD—standard deviation; QALY—quality-adjusted life year; ICER—incremental cost effectiveness ratio; ΔC = incremental cost (GEP-informed cost—standard care cost); ΔE = incremental effectiveness (GEP-informed mean QALY—standard care QALY).

## Data Availability

Patient-level administrative data used in this retrospective study are confidential and are not available in a public repository, in accordance with institutional policies.

## Appendix A

**Table A1:** Mean Cost of Treatment for Cure, Complete Response and Relapsed Disease Health States (Standard Care).

Months Following Diagnosis	Cure, Complete Response		Relapsed Disease	
	Mean	SE	Mean	SE
6	$30,425	$1543	$19,308	$2561
12	$4590	$250	$10,417	$10,530
18	$2496	$118	$7441	$10,382
24	$1870	$194	$5703	$6857
30	$1593	$161	$4495	$3904
36	$1447	$139	$3592	$2142
42	$1363	$124	$2894	$1267
48	$1314	$115	$2344	$935
54	$1287	$112	$1904	$745
60	$1274	$115	$1551	$661

Adapted from Costa et al., 2019 [22], adjusted to 2018 CAD.

**Table A2:** Mean Cost of Treatment for Cure, Complete Response and Relapsed Disease Health States (Ibrutinib).

Months Following Diagnosis	Cure, Complete Response		Relapsed Disease	
	Mean	SE	Mean	SE
6	$78,540	$1543	$67,423	$2561
12	$52,705	$250	$58,532	$10,530
18	$50,611	$118	$55,556	$10,382
24	$49,985	$194	$53,818	$6857
30	$49,708	$161	$52,610	$3904
36	$49,562	$139	$51,707	$2142
42	$49,478	$124	$51,009	$1267
48	$49,429	$115	$50,459	$935
54	$49,402	$112	$50,019	$745
60	$49,389	$115	$49,666	$661

Parametric approximations of post-relapse survival (B) are subject to issues of small sample size (*n* = 14) and the presence of a sizeable minority of individuals (*n* = 5) who did not die during the observation period. It is important to keep this limitation in mind when interpreting the results from second-line treatment.

## Appendix B. Methodological and Computational Note: Generation of Transition Probabilities from Stochastic Survival Data

Time-dependent transition probabilities were generated from patient-level time-to-event data using a three-step process.

Step 1. Time-to-event analysis using Generalized Linear Means (GLM) regression.

Time-to-event data from our retrospective cohort were analyzed using the ‘streg’ process in Stata 13. Four types of events were analyzed:

1. Time from Complete Response to Treatment Failure/Relapse
2. Time from Treatment Failure/Relapse to Death
3. Time from Complete Response to Death
4. Time from Cure to Death

The output of the ‘streg’ process is a table containing an estimate of the constant ($\beta_{o}$) and shape parameter (*ρ*), where scale parameter λ = $e^{\beta_{o}}$. An example is shown below with the relevant values indicated in italicized bold:

**Table A3:** A Stata streg output with a Weibull link.

_t	Coef.	Std. Err.	z	P > |z|	[95% Conf. Interval]	
*_cons*	*−4.861465*	*0.562269*	−8.65	0.000	−5.963492	−3.759438
*/ln_p*	*−0.5228583*	*0.* *1233935*	−4.24	0.000	−0.7647051	−0.2810115
P 1/p	0.5928236 1.686842	0.0731506 0.2081453			0.4654712 1.324469	0.7550196 2.148361

Step 2. Monte Carlo sampling of regression coefficients.

A value for log($\beta_{o}$) is sampled from a normal distribution based on the mean and standard error within the output. This produces NMC estimates of the log constant term where NMC is the number of Monte Carlo simulations performed.

To estimate treatment effect (in the GEP-informed strategy), a similar normal distribution sampling procedure was performed on the mean and standard deviation of log(HR) to produce an estimate of the treatment coefficient ($\beta_{trt}$). The sampled value of log(HR) was added to each estimate of log($\beta_{o}$), producing a predicted treatment-adjusted HR consistent with the assumptions of a Weibull GLM model: $\lambda_{trt}=e^{\beta_{o}+\beta_{trt}}$, where *λ* is the scale parameter of the Weibull distribution. For the Standard Care strategy, $\beta_{trt}$ = 0, and therefore the value of *λ* is simply $e^{\beta_{o}}$.

Step 3. Conversion of Weibull parameters to time-dependent transition probabilities.

A set of time-dependent transition probabilities can be estimated from the shape (γ) and scale (*λ*) parameters of a Weibull distribution according to the following formula: $Tp=1-e^{\lambda {\left(t-u\right)}^{\rho }-\lambda t^{\rho }}$, where *ρ* and *λ* are the shape and scale parameters of the Weibull distribution, *t* is the length of time that has occurred in the model (i.e., the number of cycles multiplied by the cycle length), and *u* is the model’s cycle length. Note that the cycle length should be expressed in the same units as the time-to-event analysis (i.e., days, months, years, etc.). This formula can be applied to the values of *ρ* and *λ* at each cycle, producing Ncycle transition probabilities, across NMC model iterations. Performing the calculation in this way preserves the nature of a gradually decreasing hazard function over time.

## References

[1] Lymphoma Canada (2017). DLBCL—Diffuse Large B Cell Lymphoma.

[2] Sehn L.H., Donaldson J., Chhanabhai M., Fitzgerald C., Gill K., Klasa R., MacPherson N., O’Reilly S., Spinelli J.J., Sutherland J. (2005). Introduction of Combined CHOP Plus Rituximab Therapy Dramatically Improved Outcome of Diffuse Large B-Cell Lymphoma in British Columbia. J. Clin. Oncol..

[3] Vose J.M., Link B.K., Grossbard M.L., Czuczman M., Grillo-Lopez A., Gilman P., Lowe A., Kunkel L.A., Fisher R.I. (2001). Phase II Study of Rituximab in Combination with CHOP Chemotherapy in Patients with Previously Untreated, Aggressive Non-Hodgkin’s Lymphoma. J. Clin. Oncol..

[4] Sehn L.H., Gascoyne R.D. (2015). Diffuse large B-cell lymphoma: Optimizing outcome in the context of clinical and biologic heterogeneity. Blood.

[5] Khor S., Beca J., Krahn M., Hodgson D., Lee L., Crump M., Bremner K.E., Luo J., Mamdani M., Bell C.M. (2014). Real world costs and cost-effectiveness of Rituximab for diffuse large B-cell lymphoma patients: A population-based analysis. BMC Cancer.

[6] Johnston K.M., Marra C.A., Connors J.M., Najafzadeh M., Sehn L., Peacock S.J. (2010). Cost-Effectiveness of the Addition of Rituximab to CHOP Chemotherapy in First-Line Treatment for Diffuse Large B-Cell Lymphoma in a Population-Based Observational Cohort in British Columbia, Canada. Value Health.

[7] Alizadeh A.A., Eisen M.B., Davis R.E., Ma C., Lossos I.S., Rosenwald A., Boldrick J.C., Sabet H., Tran T., Yu X. (2000). Distinct types of diffuse large B-cell lymphoma identified by gene expression profiling. Nature.

[8] Lenz G., Wright G.W., Emre N.C.T., Kohlhammer H., Dave S.S., Davis R.E., Carty S., Lam L.T., Shaffer A.L., Xiao W. (2008). Molecular subtypes of diffuse large B-cell lymphoma arise by distinct genetic pathways. Proc. Natl. Acad. Sci. USA.

[9] Scott D.W., Mottok A., Ennishi D., Wright G.W., Farinha P., Ben-Neriah S., Kridel R., Barry G.S., Hother C., Abrisqueta P. (2015). Prognostic Significance of Diffuse Large B-Cell Lymphoma Cell of Origin Determined by Digital Gene Expression in Formalin-Fixed Paraffin-Embedded Tissue Biopsies. J. Clin. Oncol..

[10] Scott D.W., Wright G.W., Williams P.M., Lih C.-J., Walsh W., Jaffe E., Rosenwald A., Campo E., Chan W.C., Connors J.M. (2014). Determining cell-of-origin subtypes of diffuse large B-cell lymphoma using gene expression in formalin-fixed paraffin-embedded tissue. Blood.

[11] Fu K., Weisenburger D.D., Choi W.W., Perry K.D., Smith L.M., Shi X., Hans C.P., Greiner T.C., Bierman P.J., Bociek R.G. (2008). Addition of Rituximab to Standard Chemotherapy Improves the Survival of Both the Germinal Center B-Cell–Like and Non–Germinal Center B-Cell–Like Subtypes of Diffuse Large B-Cell Lymphoma. J. Clin. Oncol..

[12] De Jong D., Rosenwald A., Chhanabhai M., Gaulard P., Klapper W., Lee A. (2007). Immunohistochemical prognostic markers in diffuse large B-cell lymphoma: Validation of tissue microarray as a prerequisite for broad clinical applications—A study from the Lunenburg Lymphoma Biomarker Consortium. J. Clin. Oncol. Off. J. Am. Soc. Clin. Oncol..

[13] Dunleavy K., Roschewski M., Wilson W.H. (2014). Precision Treatment of Distinct Molecular Subtypes of Diffuse Large B-cell Lymphoma: Ascribing Treatment Based on the Molecular Phenotype. Clin. Cancer Res..

[14] Roschewski M., Staudt L.M., Wilson W.H. (2014). Diffuse large B-cell lymphoma—treatment approaches in the molecular era. Nat. Rev. Clin. Oncol..

[15] Intlekofer A., Younes A. (2014). Precision therapy for lymphoma—current state and future directions. Nat. Rev. Clin. Oncol..

[16] Swerdlow S.H., Campo E., Pileri S.A., Harris N.L., Stein H., Siebert R., Advani R., Ghielmini M., Salles G.A., Zelenetz A.D. (2016). The 2016 revision of the World Health Organization classification of lymphoid neoplasms. Blood.

[17] Younes A., Sehn L.H., Johnson P., Zinzani P.L., Hong X., Zhu J., Patti C., Belada D., Samoilova O., Suh C. (2019). Randomized Phase III Trial of Ibrutinib and Rituximab Plus Cyclophosphamide, Doxorubicin, Vincristine, and Prednisone in Non–Germinal Center B-Cell Diffuse Large B-Cell Lymphoma. J. Clin. Oncol..

[18] Wilson W.H., Wright G.W., Huang D.W., Hodkinson B., Balasubramanian S., Fan Y., Vermeulen J., Shreeve M., Staudt L.M. (2021). Effect of ibrutinib with R-CHOP chemotherapy in genetic subtypes of DLBCL. Cancer Cell.

[19] Wright G.W., Huang D.W., Phelan J.D., Coulibaly Z.A., Roulland S., Young R.M., Wang J.Q., Schmitz R., Morin R., Tang J. (2020). A Probabilistic Classification Tool for Genetic Subtypes of Diffuse Large B Cell Lymphoma with Therapeutic Implications. Cancer Cell.

[20] Canadian Agency for Drugs and Technology in Health (CADTH) (2017). Guidelines for the Economic Evaluation of Health Technologies.

[21] Briggs A., Claxton K., Sculpher M. (2006). Decision Modelling for Health Economic Evaluation.

[22] Costa S., Scott D.W., Steidl C., Peacock S.J., Regier D.A. (2019). Real-World Costing Analysis for Diffuse Large B-Cell Lymphoma in British Columbia. Curr. Oncol..

[23] Costa S., Regier D., Meissner B., Cromwell I., Ben-Neriah S., Chavez E., Hung S., Steidl C., Scott D., Marra M. (2016). A Time-and-Motion Approach to Micro-Costing of High-Throughput Genomic Assays. Curr. Oncol..

[24] CADTH pan-Canadian Oncology Drug Review Final Economic Guidance Report (2015). Ibrutinib (Imbruvica) for Chronic Lymphocytic Leukemia or Small Lymphocytic Lymphoma. https://www.cadth.ca/sites/default/files/pcodr/pcodr-ibrutinib-cll-sll-fn-egr.pdf.

[25] CADTH pan-Canadian Oncology Drug Review Final Economic Guidance Report (2013). Brentuximab Vedotin (Adcetris) for Hodgkin Lymphoma. https://www.cadth.ca/sites/default/files/pcodr/pcodr-adcetrishl-fn-egr.pdf.

[26] CADTH pan-Canadian Oncology Drug Review Final Economic Guidance Report (2016). Lenalidomide (Revlimid) for Newly Diagnosed Multiple Myeloma. https://www.cadth.ca/sites/default/files/pcodr/pcodr_lenalidomide_revlimid_nd-mm_fn_egr.pdf.

[27] ClinicalTrials.gov (2017). Efficacy and Safety Study of Lenalidomide Plus R-CHOP Chemotherapy Versus Placebo Plus R-CHOP Chemotherapy in Untreated ABC Type Diffuse Large B-Cell Lymphoma (ROBUST). NCT02285062.

[28] ClinicalTrials.gov (2017). A Study of the Bruton’s Tyrosine Kinase Inhibitor, PCI-32765 (Ibrutinib), in Combination with Rituximab, Cyclophosphamide, Doxorubicin, Vincristine, and Prednisone in Patients with Newly Diagnosed Non-Germinal Center B-Cell Subtype of Diffuse Large B-Cell Lymphoma. NCT01855750.

[29] ClinicalTrials.gov (2015). A Study of Brentuximab Vedotin in Relapsed or Refractory Non-Hodgkin Lymphoma. NCT01421667.

[30] Knight C., Hind D., Brewer N., Abbott V. (2004). Rituximab (MabThera^®^) for aggressive non-Hodgkin’s lymphoma: Systematic review and economic evaluation. Health Technol. Assess..

[31] Doxorubicin, Cyclophosphamide, Vincristine, Prednisone and Rituximab (CHOP-R) Chemotherapy protocols—Lymphoma & Myeloma 2014. http://www.bccancer.bc.ca/chemotherapy-protocols-site/Documents/Lymphoma-Myeloma/LYCHOPR_Protocol_1Oct2014.pdf.

[32] Siebert U., Alagoz O., Bayoumi A.M., Jahn B., Owens D.K., Cohen D.J., Kuntz K.M. (2012). State-Transition Modeling: A Report of the ISPOR-SMDM Modeling Good Research Practices Task Force-3. Value Health.

[33] Statistics Canada (2017). Consumer Price Index, Health and Personal Care, by Province (British Columbia). http://www.statcan.gc.ca/tables-tableaux/sum-som/l01/cst01/econ161k-eng.htm.

[34] Staton A.D., Chen Q., Ayer T., Goldstein D., Koff J.L., Flowers C.R. (2015). Cost-Effectiveness of Subtype-Based Treatment Strategies for Diffuse Large B-Cell Lymphoma Patients (DLBCL). Blood.

[35] Chen Q., Staton A.D., Ayer T., Goldstein D., Koff J.L., Flowers C.R. (2017). Exploring the potential cost-effectiveness of precision medicine treatment strategies for diffuse large B-cell lymphoma. Leuk. Lymphoma.

[36] Weymann D., Pataky R., Regier D.A. (2018). Economic Evaluations of Next-Generation Precision Oncology: A Critical Review. JCO Precis. Oncol..

[37] Regier D.A., Veenstra D.L., Basu A., Carlson J.J. (2019). Demand for Precision Medicine: A Discrete-Choice Experiment and External Validation Study. PharmacoEconomics.

[38] Staiger A., Ziepert M., Horn H., Scott D., Barth T., Bernd H.-W. (2017). Clinical Impact of the Cell-of-Origin Classification and the MYC/BCL2 Dual Expresser Status in Diffuse Large B-Cell Lymphoma Treated Within Prospective Clinical Trials of the German High-Grade Non-Hodgkin’s Lymphoma Study Group. J. Clin. Oncol..

